# Stochastic events can explain sustained clustering and polarisation of opinions in social networks

**DOI:** 10.1038/s41598-020-80353-7

**Published:** 2021-01-14

**Authors:** Scott A. Condie, Corrine M. Condie

**Affiliations:** 1 CSIRO Oceans and Atmosphere, GPO Box 1538, Hobart, TAS 7001 Australia; 2 Centre for Marine Socioecology, University of Tasmania, Hobart, TAS Australia; 3 Institute for Marine and Antarctic Studies, University of Tasmania, Hobart, Australia

**Keywords:** Complex networks, Computer modelling

## Abstract

Understanding the processes underlying development and persistence of polarised opinions has been one of the key challenges in social networks for more than two decades. While plausible mechanisms have been suggested, they assume quite specialised interactions between individuals or groups that may only be relevant in particular contexts. We propose that a more broadly relevant explanation might be associated with the influence of external events. An agent-based bounded-confidence model has been used to demonstrate persistent polarisation of opinions within populations exposed to stochastic events (of positive and negative influence) even when all interactions between individuals are noisy and assimilative. Events can have a large impact on the distribution of opinions because their influence acts synchronistically across a large proportion of the population, whereas an individual can only interact with small numbers of other individuals at any particular time.

## Introduction

Social interactions through personal or virtual contact can influence the perceptions, opinions and beliefs of individuals^1,2^. Theories on social influence, supported to varying degrees by empirical laboratory data, have been incorporated into a range of social influence models drawing on established statistical physics methods^3–6^. While there have been renewed efforts to review and reconcile alternate models^7–13^, a unified framework linking robust predictions to alternative assumptions is yet to emerge.

Social influences are usually assimilative (Table 1A), with the opinions of individuals brought closer by factors such as convincing arguments^14^, the dominance of individuals with more knowledge or status^15^, or pressure to conform to group norms^1^. However, it has also been long recognised that differences in opinion can resist assimilative forces and persist over extended periods^3^. This can be partially explained by the concept of homophily, which describes the preference for communicating with like-minded individuals and a resulting tendency to be more strongly influenced by those individuals^2^.

**Table 1:** Social influence model types, underlying assumptions and emergent behaviours.

Model type	Underlying assumption	Emergent behaviour	Graphic summary
(A) Assimilative influence	Individuals influence each other towards reducing opinion differences	Opinions converge towards a consensus	
(B) Bounded assimilative influence	Only individuals with similar opinions influence each other (homophily)	Opinions fragment into two or more converging clusters, all within the initial range of opinions	
(C) Noisy bounded assimilative influence	As in B except that opinions also experience random fluctuations due to external influences	Opinion clusters start to form (as in B) before converging towards a broad distribution of opinions (distribution becomes more uniform as noise amplitude increases)	
(D) Differentiative influence	Individuals with very dissimilar opinions can influence each other towards even more dissimilar opinions (xenophobia)	Opinions diverge towards extremes that may fall outside the initial range of opinions	

Bounded confidence models explicitly represent the bounds placed on assimilative influence by homophily and successfully predict fragmentation of opinions into clusters (Table 1B). However, this fragmentation cannot be sustained in the presence of random fluctuations (noise) in opinions due to stochastic external factors. Such fluctuations will always be present in any realistic social network and allow opinions to occasionally move inside the homophily influence limit, resulting in a gradual convergence of opinion clusters^16^ (Table 1C). While large amplitude fluctuations block this convergence, they also tend to promote extreme individualism that prevents clustering. Time-varying clustering can be perpetuated by including a feedback, whereby the amplitude of fluctuations increases with the size of clusters^17^. However, this desire for individualism may only be applicable to specific issues or circumstances.

One of the most important types of fragmentation is the polarisation of opinion towards more extremist viewpoints. Polarisation has become increasingly problematic over the past decade, driving community conflict around many social and environmental issues^18–20^, as well as political extremism motivating activism and sometimes even violence^21,22^. Because extremist views sometimes lie outside the initial range of opinions, they cannot be explained by homophily and bounded confidence alone. This led to the suggestion that influences can be differentative (repulsive), whereby individuals influence each other towards more dissimilar opinions^23,24^ (Table 1D). However, the empirical evidence for differentative influence is far from conclusive^25^. An alternative mechanism has been proposed whereby intensifying discussion between like-minded individuals moves their common view towards a more extreme position^14^, sometimes referred to as bandwagoning or group-shifting^26,27^. Both mechanisms may well influence highly engaged individuals and lead to polarisation around emotive issues^28^, particularly when engagement is through social media. However, amongst the broader community, there is limited opportunity for either of these mechanisms to operate effectively^23^.

Here we propose a potentially more general hypothesis to explain both the perpetuation of opinion clustering and development of extreme opinions. We show that these behaviours tend to emerge when individuals are influenced, not only by other individuals, but also by influential events^29,30^. This differs fundamentally from suggested random changes in the opinions of individuals^31^ in that events are capable of influencing many individuals synchronistically over a limited period of time. Synchronicity across a broad community helps promote collective behaviour, potentially resulting in group-shifts towards more extreme viewpoints^26^.

## Methods

The proposed Social Influence and Event Model (SIEM) builds on the Hegselmann-Krause (HK) bounded confidence model^4^. The HK model^4^ was selected over the other seminal Deffuant–Weisbuch (DW) model^32^ (which treats pair-wise interactions sequentially) because of the need to synchonise influences of individuals and events. It represents a network of individuals (or groups) with potential to influence each other’s opinions in relation to a specific issue. Opinions of individuals are continuous (rather than being limited to discrete choices^33^) and individuals influence each other simultaneously at each timestep. Influence was assumed to be assimilative and could only occur between (randomly) linked individuals whose opinions were not too far from each other (i.e. homophily).

The opinion of individual $i$ at timestep $t + 1$ is given by:

1 $$O_{i,t + 1} = \mathop \sum \limits_{j \in I} w_{ij,t} O_{j,t} ,$$

where $I$ is the set of individuals with which $i$ interacts (including $i$ itself) and $w_{ij,t}$ is a weighting for the influence of individual $j$ on individual $i$ (all model variables are also defined in Table 2). For simplicity, an individual’s own opinion has been weighted equally to others. However, egocentric discounting of other opinions^34^ is implicit in the representation of bounded confidence. The bounded confidence assumption is:

2 $$I = \left\{ {j | \left| {O_{i,t} - O_{j,t} } \right| < \varepsilon } \right\},$$

where $\varepsilon$ is usually referred to as the confidence threshold^4,10^ and is a characteristic of the social network (rather than varying between individuals). The model also included random fluctuations (noise) in the opinions of individuals that allowed opinions to drift and occasionally move inside the confidence threshold of other individuals. In the absence of other influences, these random fluctuations ultimately drive convergence of opinion clusters that may initially form due to homophily^16^.

**Table 2:** Definitions of model variables describing both individual and system characteristics.

Variable	Definition	Range
**Individual characteristics**		
$O_{i,t}$	Opinion of individual $i$ at timestep $t$	$\left[ { - 1 \,\,1} \right]$
$w_{ij,t}$	Weighting for the influence of individual $j$ on individual $i$ at timestep $t$	$\left[ {0\,\, 1} \right]$
$C_{i,t}$	Certainty of individual $i$ in their opinion at timestep $t$	$\left[ {0\,\, 1} \right]$
**System characteristics**		
*k*	Average number of links per individual (not all influential)	
$\varepsilon$	Confidence threshold (defining level of homophily)	$\left[ {0\,\, 1} \right]$
$E_{s,t}$	Strength of an event occurring at timestep $t$	$\left[ {0 \,\,1} \right]$
$E_{f}$	Mean frequency of events (probability of an event within timestep)	$\left[ {0\,\, 1} \right]$
$E_{d}$	Mean duration of events	$\left[ {0 } \right.\,\,\left. \infty \right)$
$\Delta O_{t}$	Conflict level at timestep $t$ (standard deviation of $O_{i,t}$)	$\left[ {0\,\, 1} \right]$

Traditionally, bounded confidence models have assumed that all individuals are identical in their potential to influence other individuals, such that $w_{ij,t} = 1/\left| I \right|$. Here we relax this assumption by considering the relative ranking of individuals in terms of their ability to influence others, which we specify in terms of the *certainty* in their opinion^35^. Certainty recognises differences not only in individual’s experiences, knowledge and access to relevant information^35^, but also personal characteristics such as persuasiveness, social status, open-mindedness and self-belief^1^. While model formulations vary, similar quantities are sometimes referred to as the influence strength or confidence of individuals^36,37^ (not to be confused with the confidence interval which is a property of the entire network system, Table 2). Ranking of certainty is consistent with experimental findings indicating that influential individuals also tend to be less susceptible to the influence of others^38^. An individual $i$ will only change their opinion if their certainty, $C_{j,t} \in \left[ {0\, 1} \right]$, is less than the average certainty of other individuals with which they interact at time *t*. This can be expressed as:

3 $$w_{ij,t} = \left\{ {\begin{array}{*{20}c} {\frac{1}{\left| I \right|} if C_{i,t} \le \frac{1}{\left| I \right|}\mathop \sum \limits_{j \in I} C_{j,t} } \\ {0 if C_{i,t} > \frac{1}{\left| I \right|}\mathop \sum \limits_{j \in I} C_{j,t} ,} \\ \end{array} } \right.$$

which defaults to $w_{ij,t} = 1/\left| I \right|$ when all individuals have equal certainty. While Eq. (3) provides a straight forward extension of the traditional HK model^4^, an equivalent formulation could be achieved by using the default weighting and expanding Eq. (2) to include certainty-based restrictions on $I$; or by allowing confidence thresholds to vary across the population^5^ (i.e. $\varepsilon = \varepsilon \left( {C_{i,t} ,C_{j,t} } \right)$).

The key innovation in SIEM is representation of external events that can influence opinions. These could be major events, such as political upheavals or natural disasters, or events of more localised significance, such as release of new information or a change in legislation. An event may attract or repulse individuals towards a particular opinion, but either way the event can be considered to be aligned to an opinion (effectively the opinion of the event). Similarly, depending on the size and nature of the event, it will have a characteristic influencing strength (effectively the certainty of the event). This formulation allows model events to influence individuals in a similar way to how individuals influenced each other. Specifically, Eq. (1) was expanded to include the influence of events:

4 $$O_{i,t + 1} = \mathop \sum \limits_{j \in I} w_{ij,t} O_{j,t} + w_{i,t} E_{s,t} ,$$

where $E_{s,t} \in \left[ {0\,\, 1} \right]$ is the strength of an event occurring at timestep $t$. The weighting for the influence of an event on individual $i$ is given by:

5 $$w_{i,t} = \left\{ {\begin{array}{*{20}l} {W\quad if\,\left| {O_{i,t} - E_{s,t} } \right| < \varepsilon \, and\, C_{i,t} \le E_{s,t} } \\ {0\quad otherwise , } \\ \end{array} } \right.$$

where $W \in \left[ {0\,\, 1} \right]$ is selected randomly for each individual at every timestep. A random value for the weighting function allows for stochastic variability in the influence of any event across the population. Equation (5) could be made more general by defining independent measures for the effective opinion and effective certainty of events. However, given that they are likely to be highly correlated, both aspects have been represented here by $E_{s,t}$.

Events were applied stochastically in the model with a mean frequency $E_{f} \in \left[ {0\,\, 1} \right]$ (equal to the probability of an event starting within any timestep) and a mean duration $E_{d}$, with individual events allocated randomly within the range $E_{d,t} \in \left[ {0 \,\,2E_{d} } \right]$. The strength of events also varied stochastically around a mean value $E_{s} \in \left[ {0.2\, \,0.8} \right]$ with $E_{s,t} \in \left[ {E_{s} - 0.2\,E_{s} + 0.2} \right]$.

Events differed from individuals in two key aspects: (i) events could potentially influence the entire population (within the constraints imposed by Eq. 5), whereas individuals could only interact with a relatively small number of other individuals within any model time-step; and (ii) events occurred stochastically and had limited duration that also varied stochastically, whereas all individuals persisted and interacted throughout the simulation. Point (ii) also distinguishes events from external information acting as a static influencing agent^39,40^.

The model consisted of a network of 490 individuals, although tests using 14–980 individuals indicated that results are insensitive to population size. The only characteristic used to differentiate individuals was their certainty. 70 individuals were randomly allocated to each of 7 overlapping certainty ranges of equal width (0.0–0.4, 0.1–0.5, 0.2–0.6, 0.3–0.7, 0.4–0.8, 0.5–0.9, 0.6–1.0). This allocation yielded a normal distribution of certainty across the population, with a mean value of 0.50 and a standard deviation of 0.25. The certainty of individuals was varied randomly at each timestep, while also being continuously attracted towards its initial value $C_{i,0}$. This constraint on the range of an individual’s certainty through time reflects its dependence on relatively stable personal characteristics (knowledge, persuasiveness, social status, open-mindedness and self-belief).

**Figure 1:**
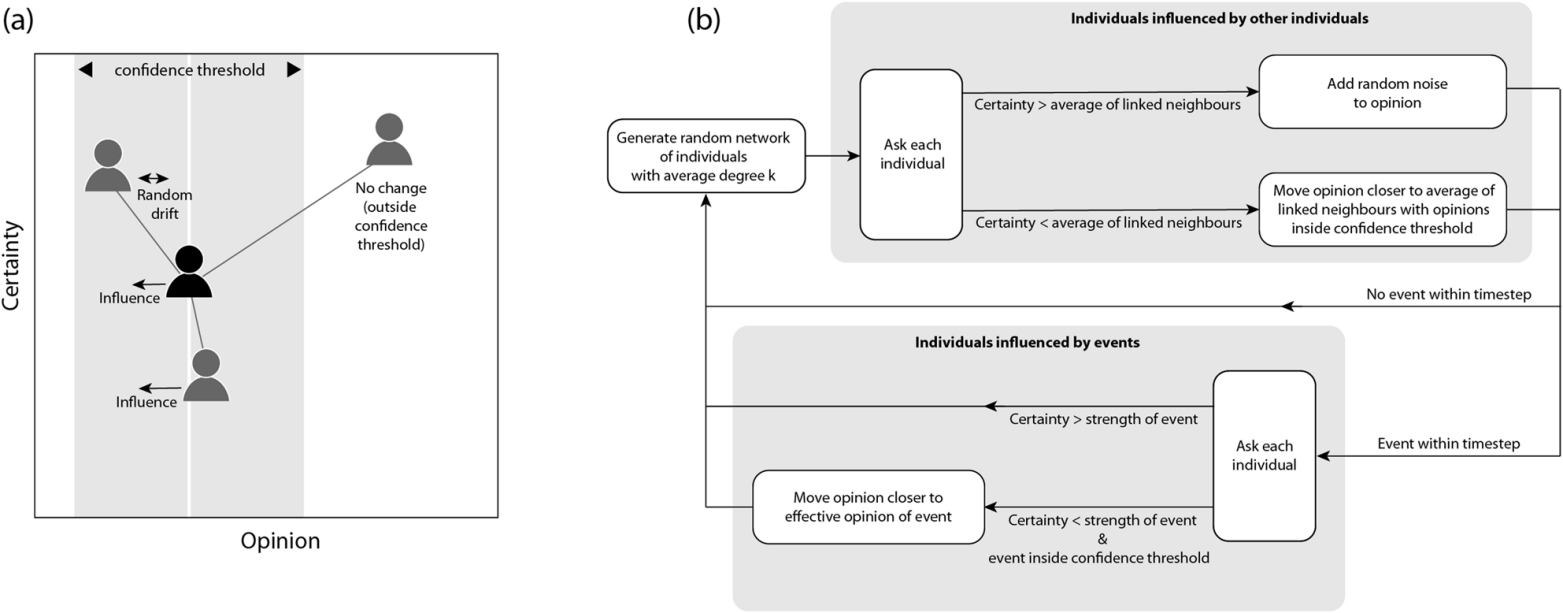
(**a**) Conditions under which individuals are influenced; and (**b**) workflow within each model timestep. Note that homophily is a system characteristic and hence confidence threshold is the same for all individuals, whereas certainty varies between individuals.

At each model timestep, opinions of individuals were influenced according to the opinions and certainties of their linked neighbours (Fig. 1a) by following a decision tree (Fig. 1b):

1. A new random network was generated with each individual forming a node with the average degree (number of links per node) being *k*.
2. Where an individual’s certainty was greater than the average of their linked neighbours, their opinion changed by a random drift $\left[ {0\,0.1\varepsilon } \right]$ that was always small compared to the confidence threshold.
3. Where an individual’s certainty was less than the average of their linked neighbours, their opinion was replaced by the average of their own opinion and those of all linked neighbours within their confidence threshold ($I$).
4. The certainty of all individuals was moved closer to their initial value by a random amount $\left[ {0 \left( {C_{i,0} - C_{i,t} } \right)} \right]$ and then a small random drift was also applied [0 0.1].

When an event occurred (or continued from the previous timestep):

1. Where an individual’s certainty was greater than the strength of an event or the event fell outside their confidence threshold, the event had no influence on their opinion.
2. Where an individual’s certainty was less than the strength of an event and the event fell inside their confidence threshold, their opinion moved closer to the event by a random amount $\left[ {0 \left( {E_{s,t} - O_{i,t} } \right)} \right]$.

Results have been described in terms of the development of the distribution of opinions across the population through time. The width of this distribution provides a simple measure of the conflict in the population, which we define here as the standard deviation in population opinions: $\Delta O_{t} = SD\left( {O_{i,t} } \right)$. Conflict levels reached a statistically steady state within 500 timesteps, after which the long-term mean was stable and repeatable. For example, when runs were repeated 10 times or runtime was extended 10 times, ensemble means always fell within one standard deviation of the mean based on any single run. Means and variances were also insensitive to population sizes for tests ranging from 14 to 980 individuals (i.e. 2.9–200% of the default population).

## Results

**Figure 2:**
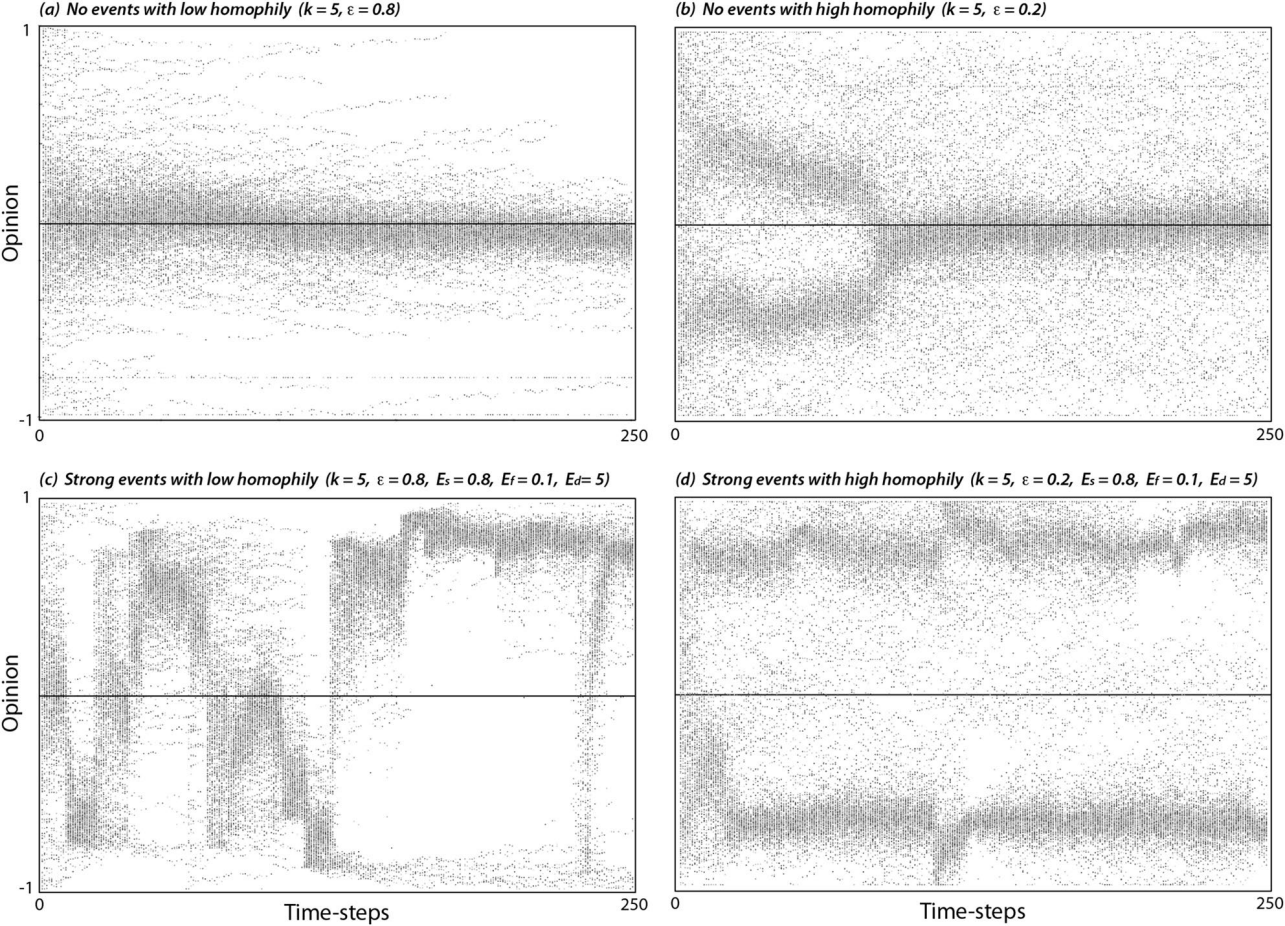
Opinions of individuals starting from a random distribution [− 1 1] under a range of conditions (first 250 timesteps shown).

**Figure 3:**
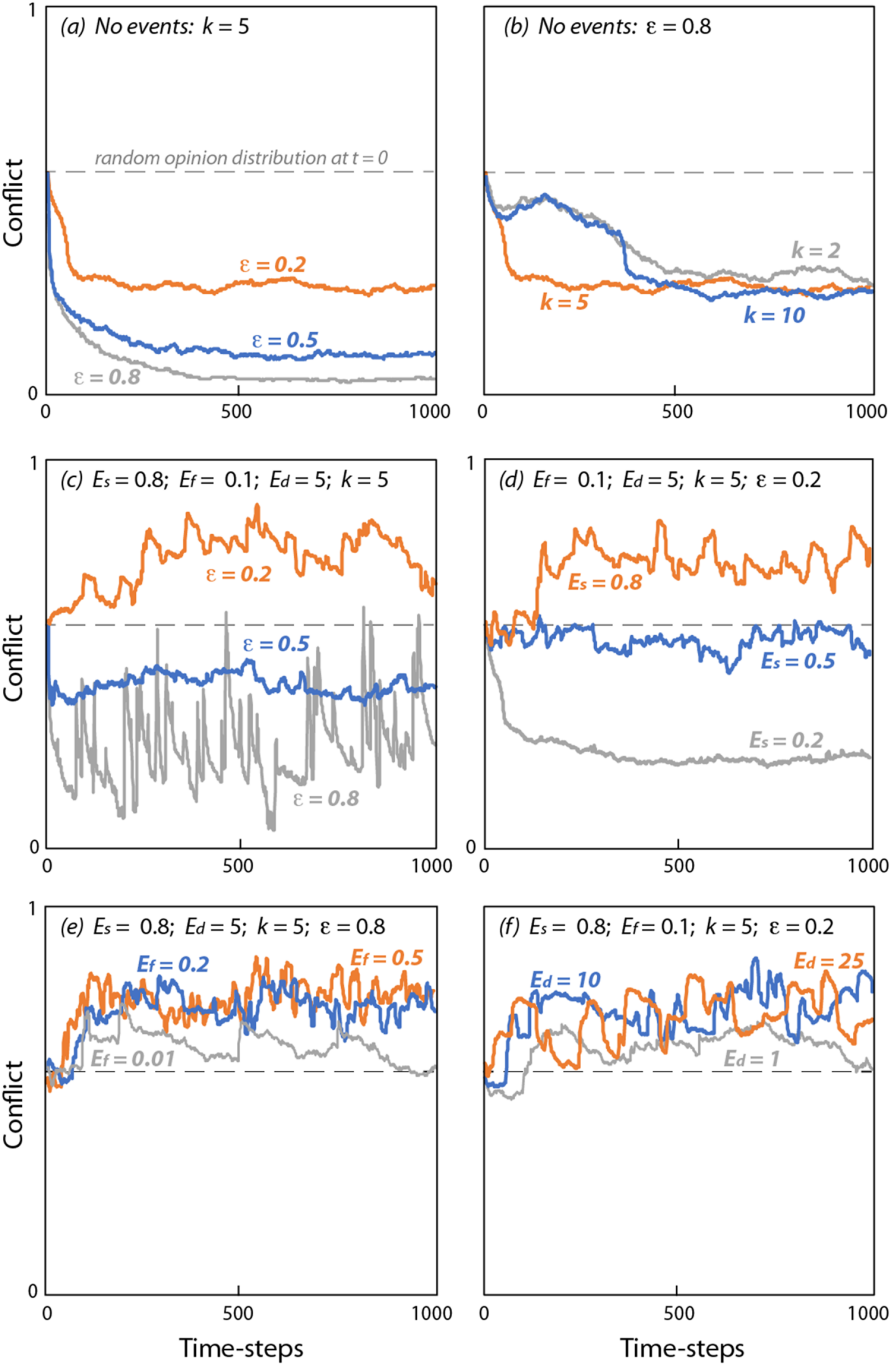
Conflict ($\Delta O_{t}$) tracked over 1000 timesteps, starting from a uniform random distribution of opinions ($\Delta O_{0} = 1/\sqrt 3 = 0.577$). Shown are dependencies on: (**a**) confidence threshold when there were no events; (**b**) average number of connections per individual per timestep when there were no events; (**c**) confidence interval when there were events; (**d**) event strength; (**e**) event frequency; and (**f**) event duration.

SIEM exhibited the range of behaviours generated by other influence models under differing levels of homophily, including both assimilative influence leading to consensus (Fig. 2a), and bounded assimilative influence leading to initial fragmentation that diminishes over time due to random fluctuations in opinions and certainty (Fig. 2b). These trends were also reflected in conflict levels, which declined rapidly under low homophily, or were temporarily sustained under high homophily before population opinions suddenly converged (Fig. 3a). Because convergence was triggered by random noise in the opinion of individuals, its timing was highly variable (Fig. 3b). Over the long-term, conflict levels increased with homophily (Fig. 3a), whereas the average number of connections per individual had a much smaller effect (Fig. 3b).

Large stochastic events ($E_{s} \ge 0.5$) generated two types of behaviour dependent on the homophily level. When homophily was low, the opinions of most of the population responded to every event, causing opinions to swing between extremes (Fig. 2c). Associated conflict levels were also highly variable, although the long-term average remained relatively low (Fig. 3c). When homophily was high, opinions diverged and became more extreme (Fig. 2d). Importantly, this polarisation was sustained as long as there were stochastic events. Average conflict levels again scaled with homophily and were sustained at high levels (Fig. 3d). Conflict was only weakly dependent on the frequency (Fig. 3e) and duration (Fig. 3f) of events, with no detectable effect once events became quasi-continuous ($E_{f} E_{d} \ge 1$).

**Figure 4:**
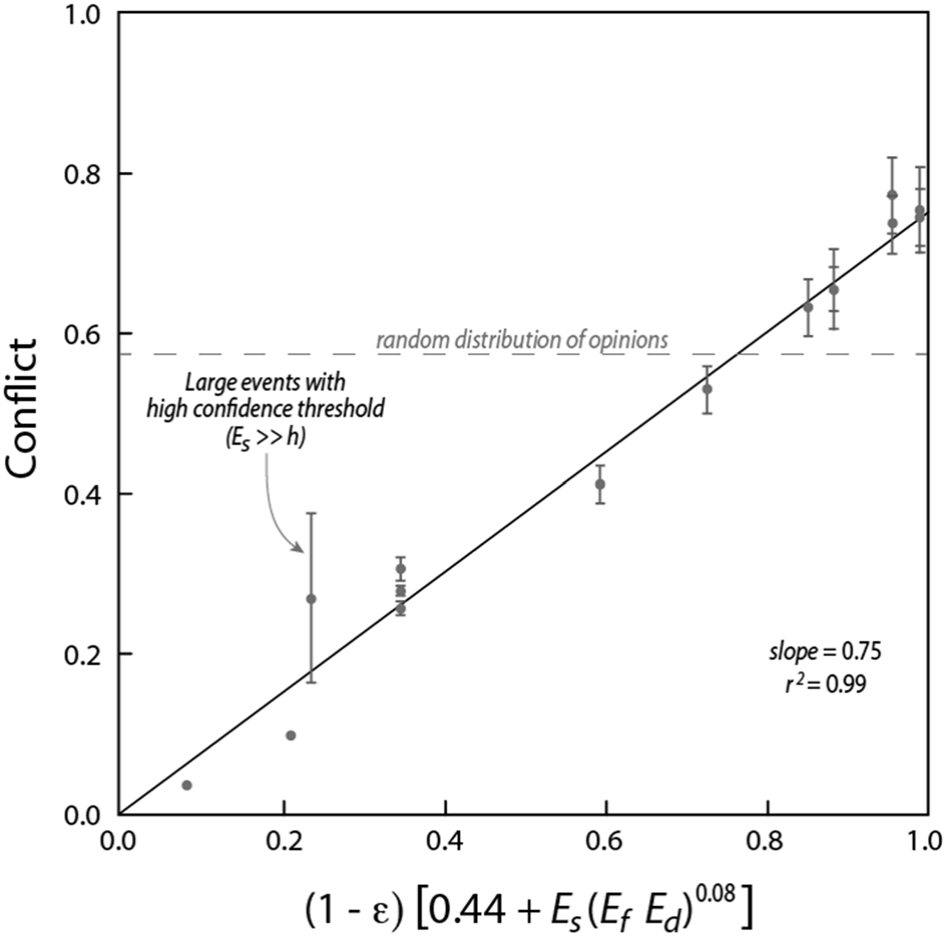
Average population conflict calculated from model results (over the period *t* = 500–1000 timesteps) plotted against the functional relationship defined by Eq. (6). The correlation was significant (*r* = 0.99, *N* = 17, *p* < 0.001) with a slope of 0.75.

Data from Fig. 3 is consistent with a linear dependency of long-term average conflict (timesteps 500–1000) on both homophily (Fig. 3c) and event strength (Fig. 3d). Whereas dependencies on event frequency (Fig. 3e) and duration (Fig. 3f) are much weaker and can be described by a simple power law for the dimensionless quantity $E_{f} E_{d}$. These dependencies can be combined into a single empirical relationship:

6 $$\Delta O = 0.75\left( {1 - \varepsilon } \right)\left[ {0.44 + E_{s} \left( {E_{f} E_{d} } \right)^{0.08} } \right]$$

which is supported by a high level of correlation (*r* = 0.99, *N* = 17, *p* < 0.001, Fig. 4). Because of the challenges associated with quantifying variables such as confidence threshold and event certainty, the values of the coefficients in Eq. (3) are less important than the structure of the relationship. Key aspects of the relationship are: (i) in the absence of events, conflict increases linearly with confidence threshold (while still being limited to relatively low levels); (ii) the effect of events on conflict increases with the product of confidence threshold and event certainty; and (iii) conflict is only weakly dependent on the frequency and duration of events. While completely removing the dependence on $E_{f} E_{d}$ (by assuming $\left( {E_{f} E_{d} } \right)^{0.08} = 1$) results in only a minor deterioration in the correlation (*r* = 0.98, *N* = 17, *p* < 0.001), its retention ensures a smooth transition from infrequent events of short duration ($E_{f} E_{d} \ll 1$) to no events ($E_{f} E_{d} = 0$).

## Discussion

Polarisation, including the emergence of views more extreme than initially present, has previously been explained by either differentative (repulsive) interactions, whereby opposing individuals influence each other towards more dissimilar opinions^23,24^; or by intensifying discussion between like-minded individuals encouraging adoption of more extreme views^14^. Both mechanisms rely on quite specific forms of interaction that have limited supporting empirical evidence outside of social media^25,26,41^. In contrast, most contested issues are influenced by events that are external to the individuals themselves^30^. These can be major events capable of influencing opinions on national or international issues, such as natural disasters, catastrophic accidents, or socio-political upheaval^42–44^. Alternatively, opinions on local issues may be influenced by smaller events, such as news releases, political decisions, or legal rulings^45,46^. In the current context, the key characteristic of any event is that it is time-bounded and can influence many individuals synchronistically.

Strong events were found to generate and indefinitely sustain polarised opinions (high conflict) among populations with relatively small confidence thresholds due to homophily (Figs. 2d and 3c). Larger confidence thresholds ($\varepsilon > 0.5$) resulted in reduced conflict levels, although still well above those where no events were present. Relatively high conflict levels (above a random distribution of population opinions) were maintained even when events were rare ($E_{f} E_{d} \ll 0$) (Fig. 3e,f). Overall, stochastic events were very effective in polarising population opinions and maintaining conflict. The net long-term effect of homophily and stochastic events on conflict in the model were well described by Eq. (6).

Equation (6) is not dependent on the distribution of certainty across individuals and, indeed, mean conflict levels do not change significantly when all individuals are given equal certainty. However, with uniform certainty, distributions of opinions at any time are essentially binary with individuals on each side of the polarisation having near identical opinions. This is clearly less realistic than the more continuous distribution of opinions evident in Fig. 2. Consideration of individual certainty will become even more important as influence models begin to resolve differences in the demographic characteristics and the vested interests of individuals and groups^38,47^.

The polarising effect of events is partly of concern because some events are not random, but rather are constructed or distorted for financial, political, or ideological reasons. These ‘hoax’ events are often labelled as crises or emergencies by their proponents^48^ and may be designed to damage individuals or organisations^49,50^, gain popular support^51^, divert scrutiny^52,53^, or justify otherwise unpopular actions^54^. Our results suggest that conflicts might be perpetuated indefinitely if one or both sides of an issue can orchestrate influential events or otherwise maintain a perception of crisis.

While it is the conceptual and structural aspects of the model that are critical to the current study, the longer-term objective for influence modelling should be its application to real social systems^55^. This transition will pose significant challenges related to the parameterisation and calibration of the model. Because all aspects of the model are expressed in relative units, relationships such as Eq. (6) are of limited practical use unless key factors, such as the influence threshold of the network and the strength of events, can be calibrated relative to the opinion and certainty scales. If the temporal evolution of opinions is of interest, then the timestep will also need to relate to real time, with frequencies and average durations of events set accordingly (Supplementary Information).

These aspects can be illustrated using the example of effects of extreme weather events in the United States influencing opinions relating to climate change. In 2012, Weather Forecasting Offices across the United States recorded event frequencies ($E_{f}$) ranging from 0 to 22 per month^56^. Comparisons of the timing of extreme weather events with climate opinions from national surveys indicate the duration ($E_{d}$) of a statistically significant influence from these events was only around 0.1 months^56,57^, implying that $\left( {E_{f} E_{d} } \right)^{0.08} <$ 1.2. Being a particularly contentious issue in the United States^18^, we can safely assume that homophily is relatively high with respect to opinions on climate change (say $\varepsilon \approx$ 0.2). While the size of extreme weather events ($E_{s}$) in terms of their potential influence on opinions is difficult to estimate from the available data, Eq. (6) suggests that values as low as 0.2 could contribute to conflict levels (${\Delta }O \approx$ 0.4 compared to ${\Delta }O \approx$ 0.26 in the absence of events). This can be compared with the statistical analysis of the national survey responses (130,000 individuals) indicating that the influence on people experiencing the highest rate of extreme events was larger than any demographic effects, although still much smaller than the effects of partisan identification (Republican or Democrat)^56^. If the size of extreme events continues to increase, the model suggests that their contribution to conflict levels may become more significant. For example, values around $E_{s} \approx$ 0.5 yield conflict levels around ${\Delta }O \approx$ 0.6 (Eq. 6), which is above randomly distributed opinions and indicative of strong polarisation.

While the example above helps demonstrate the potential relevance of social influence models to contested issues such as climate change, more targeted data collection coupled with rigorous calibration procedures may in future provide powerful tools able to test a wide range of strategies aimed at reducing conflict within and across communities.

## Supplementary Information

Supplementary Information.

## Data Availability

The model output dataset generated during the current study is provided as Supplementary material.
